# From Sound to Silence: Cerebellar Abscesses and Herniation Due to Cochlear Implant Infection With *Escherichia coli* and *Bacteroides fragilis*

**DOI:** 10.1155/crdi/5645503

**Published:** 2026-01-06

**Authors:** Celine Molfetta, Ruchi Biswas, Marc Pickard, Tanzila Salim, Carlos Nuñez

**Affiliations:** ^1^ Division of Infectious Diseases, Rutgers New Jersey Medical School, Newark, New Jersey, USA, rutgers.edu

## Abstract

Cochlear implantation (CI) is a safe and well‐established intervention for sensorineural hearing loss, with a low incidence of severe postoperative infections. We present the first reported case of cerebellar abscess and herniation due to CI infection. This unique case involves a 57‐year‐old man with recurrent cochlear implant infections, necessitating multiple debridements and eventual removal of the implant body while retaining the electrode array. This ultimately led to the formation of two right‐sided cerebellar abscesses. His course was further complicated by cerebellar herniation due to mass effect, requiring urgent neurosurgical intervention and extensive antimicrobial therapy. Despite the grave prognosis, timely intervention led to significant clinical improvement. This case highlights the pathophysiology, implicated organisms, and management of cerebellar abscesses following CI/explantation, underscoring the importance of early recognition, aggressive infection control, and multidisciplinary management in this rare but serious complication.

## 1. Introduction

Cochlear implantation (CI) remains the mainstay of intervention for sensorineural hearing impairment in children and adults, with a low 4% estimated incidence of postoperative infection. Though rare, studies report a 3% rate of major adverse infectious complications necessitating surgical intervention [1]. Infection is commonly a result of impaired wound healing (*Staphylococcus aureus* most frequently reported perioperatively), acute otitis media, and chronic ear pathology. The majority of postoperative infections present with a time lag, commonly meningitis, tinnitus, cerebrospinal fluid (CSF) otorrhea, and rarely, abscess formation [1, 2]. Reported cases include retroauricular and subperiostal abscess formation, highlighting infiltration of local structures [3]. Eggink et al. suggest that otogenic infections may extend directly through the compromised dural plate, or through the labyrinth and cochlear aqueduct [4]. Despite this, intracranial, specifically cerebellar, extension is exceedingly rare. Treatment includes site irrigation, systemic antibiotics, surgical debridement, and possible contralateral CI [2].

Cerebellar abscess formation is extremely rare, with an estimated prevalence of 0.3–0.9 per 100,000 population per year, with high morbidity/mortality. It is commonly secondary to acute mastoiditis or acute otitis media [5]. Symptoms include fever, nausea, vomiting, gait ataxia, dysmetria, nystagmus, vertigo, headache, seizure, altered mentation, or weakness. Cerebellar abscesses causing mass effect may have grim impending consequences, such as herniation. Alarm symptoms and signs include papilledema, pupillary asymmetry, posturing, respiratory distress/arrest, and other neurological deficits, dependent upon affected anatomic structures [6].

Here, we present the rare, first‐reported case of cerebellar abscess formation from right‐sided CI infection, resulting in cerebellar herniation and infarcts.

## 2. Case Presentation (Brief Summary, Figure 1)

### 2.1. Initial Surgery and Postoperative Infections (2022‐2023)

A 57‐year‐old man with a history of a right vestibular schwannoma underwent translabyrinthine resection with cochlear implant placement in 2022. His postoperative course was complicated by recurrent surgical‐site infections characterized by pain, swelling, and purulent drainage. He required multiple incisions and drainage procedures, surgical revisions, and courses of antimicrobial therapy for hardware infections involving *Escherichia coli*, *Pseudomonas aeruginosa*, and *Candida parapsilosis*.

**Figure 1:**
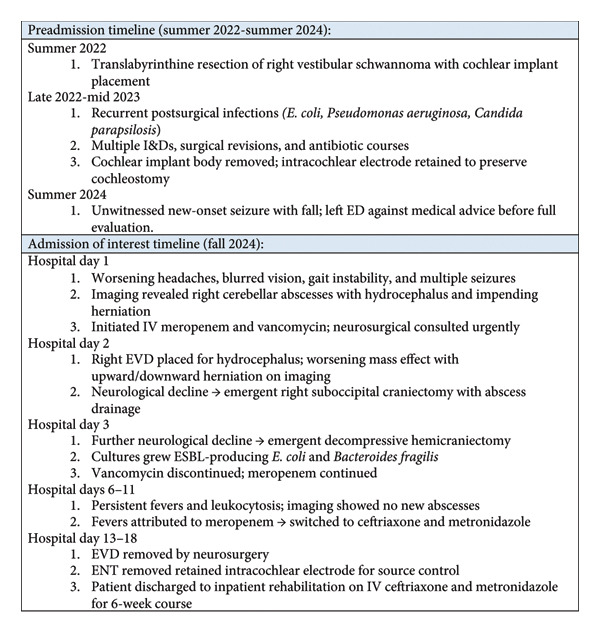
Brief bullet point summary of clinical course and key events.

In the summer of 2023, the cochlear implant body was removed, while the electrode array was left in place to preserve the cochleostomy for potential reimplantation.

### 2.2. First Neurological Event (Summer 2024)

In late summer 2024, the patient presented with a first‐time seizure. Neurology and Infectious Diseases were consulted for evaluation of possible meningoencephalitis or toxic encephalopathy. EEG showed no epileptiform activity, but the patient left against medical advice before further diagnostic workup could be completed.

### 2.3. Presentation and Initial Findings (Fall 2024)

He returned in the fall of 2024 with worsening headaches, blurry vision, fevers, gait instability with frequent falls, and nine seizures over two months.

On arrival, he was hypertensive (155/90), afebrile, and oxygenating well on room air. He was alert and oriented but exhibited right‐sided deficits, including decreased sensation, dysmetria, dysdiadochokinesia, and horizontal gaze nystagmus. There was no skin breakdown, otorrhea, otalgia, or mastoid tenderness. Blood and CSF cultures were negative (Table 1).

**Table 1:** Notable laboratory data throughout patient’s hospitalization.

Cell blood count	Day 1	Day 2	Day 3	Day 6	Day 11
WBC (×10^9^ μL)	7.7	6.8	9.2	**15.3 (H)**	9.5
Neutrophil (absolute) (×10^3^/μL)	5.5	3.8	7.4	13.4	
Lymphocyte (absolute) (×10^3^/μL)	1.3	2.0	0.9	0.5	
Monocyte (absolute) (×10^3^/μL)	0.8	0.9	0.9	1.1	

*Microbiology*					
Blood culture	No growth after 6 days			No growth after 5 days	
Abscess culture			**Many *E. coli***		
Anaerobic abscess culture			***Bacteroides fragilis*** Beta lactamase positive		
Acinetobacter screen rectal culture		Not detected			
Carbapenemase rectal screen		**ESBL *E. coli***; no carbapenemase‐producing isolate			
*Candida auris* rectal swab		Not detected			
CSF culture			No growth	No growth	
Fungus culture			No fungus isolate after 4 weeks		
MRSA nasal swab		Not detected			

*Antibiotics*					
	IV Meropenem and vancomycin initiated		IV Vancomycin discontinued. IV meropenem continued		IV Meropenem discontinued. IV ceftriaxone and metronidazole initiated

*CSF tests*					
CSF glucose (mg/dL)			71	**105 (H)**	
CSF protein (mg/dL)			**10 (L)**	20	
CSF appearance			**Slightly cloudy**	Clear	
CSF RBC count (c/μL)			**909 (H)**	**767 (H)**	
CSF WBC count (c/μL)			**48 (H)**	0	

*Note:* The bold values represent “flagged” or “abnormal” results.

**Figure 2:**
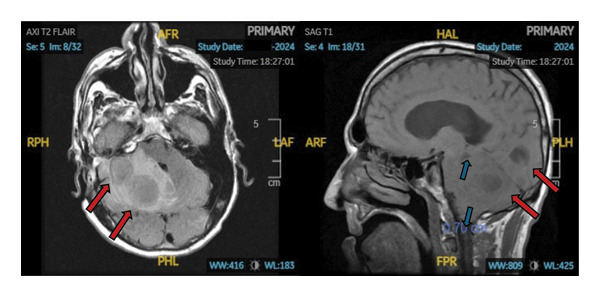
MRI of the brain on admission. Images show two peripherally enhancing masses in the right cerebellar hemispheres, with the lateral lesion measuring up to 2.6 cm and the medial lesion measuring up to 3.3 cm (red arrows). There is also significant mass effect with downward (cerebellar tonsils lie 7 mm below the foramen magnum on the right and 6 mm on the left) and upward herniation, effacement of the cerebral aqueduct, and acute noncommunicating hydrocephalus (blue arrows).

CT and MRI of the brain revealed two adjacent right cerebellar abscesses (2.6 and 3.3 cm), causing significant mass effect with impending upward and downward herniation, effacement of the cerebral aqueduct, and acute communicating hydrocephalus (Figure 2).

### 2.4. Initial Management

Empiric intravenous (IV) antibiotics were initiated with meropenem (2 g every 8 h) for broad‐spectrum central nervous system coverage, including multidrug‐resistant gram‐negative aerobes and anaerobes, and vancomycin (20 mg/kg loading dose followed by 15 mg/kg every 8 h) for resistant gram‐positive cocci, particularly MRSA.

Neurosurgery was urgently consulted. The following day, the patient developed worsening headaches and emesis, though his neurological exam remained stable. Due to concerns for increased intracranial pressure (ICP), a right external ventricular drain (EVD) was placed for CSF diversion and ICP monitoring. Subsequent imaging showed worsening hydrocephalus and evidence of upward transtentorial and downward tonsillar herniation (Table 2).

**Table 2:** Diagnostic impressions of patient’s CT head scans throughout the hospital course, highlighting key changes and findings at various stages of treatment.

Day 1 17:05	Day 2 22:57	Day 3 09:13	Day 3 15:07
1. Right cerebellar and vermis edema with partial effacement of the fourth ventricle, prepontine cistern, and interpeduncular cistern 2. Rounded hypodensities in the right cerebellum consistent with abscesses 3. Impending upward/downward herniation 4. Noncommunicating hydrocephalus with lateral and third ventricle enlargement	1. Right frontal ventriculostomy catheter placed 2. Worsening obstructive hydrocephalus 3. Severe posterior fossa mass effect with downward tonsillar and upward transtentorial herniation	1. Worsening nonobstructive hydrocephalus and cortical sulcal effacement 2. Stable but severe mass effect with downward/upward herniation	1. Status/postsuboccipital craniotomy and abscess drainage 2. Decreased mass effect with decreased effacement of the perimesencephalic and basal cisterns, cortical sulci, and hydrocephalus 3. Persistent downward/upward herniation and cerebellar sulcal effacement

**Day 4 00:46**	**Day 4 17:27**	**Day 9 09:09**	

1. Increased mass effect and effacement of the basal cisterns, cortical sulci with obstructive hydrocephalus 2. Stable upward/downward herniation 3. Enlarged lateral and third ventricles with debris in the occipital horns, suggestive of ventriculitis	1. Status/postdecompressive suboccipital craniectomy/resection of cerebellar abscesses 2. Persistent noncommunicating hydrocephalus with a stable ventricular size and a right ventriculostomy catheter in place	1. New bilateral cerebellar infarcts superimposed on evolving right‐sided edema, maintaining posterior fossa mass effect with upward/downward herniation 2. Right ventriculostomy with decreased ventricular size and reduced transependymal CSF flow	

Several hours later, the patient became less responsive and was taken emergently to the operating room by neurosurgery for a right suboccipital craniectomy with drainage of the two cerebellar abscesses. The retained electrode array was not removed at this time, with plans for future removal by otolaryngology (ENT).

### 2.5. Clinical Deterioration and Antimicrobial Adjustments

On Postoperative day 1, his condition deteriorated with increased lethargy, inability to follow commands, pinpoint pupils with upward gaze preference, and irregular respiration. An emergent suboccipital decompressive hemicraniotomy was performed, resulting in clinical improvement.

Intraoperative cultures grew rare *E. coli* and *Bacteroides fragilis* (Table 1). Rectal swab confirmed extended‐spectrum beta‐lactamase (ESBL)–producing *E. coli* (Table 1). Vancomycin was discontinued, and meropenem was continued due to its efficacy against both ESBL‐producing *Enterobacterales* and anaerobic organisms (*Bacteroides fragilis*). Repeat head CT showed new bilateral cerebellar infarcts and decreased ventricular size (Table 2).

On Hospital day 6, the hospital course was further complicated by persistent fevers and leukocytosis (Table 1). His surgical site remained clean, dry, and intact. Repeat blood and CSF cultures were negative (Table 1). Sputum cultures grew *Candida albicans*, deemed likely to be colonization (Table 1). CT of abdomen and pelvis demonstrated patchy dependent bilateral opacities in the lung, bilateral pleural effusions, ascites, esophagitis, and air within the bladder, suggestive of a gas‐producing infection, but urine analysis was negative for nitrites or leukocyte esterase.

Fevers persisted despite clinical improvement and resolution of leukocytosis, raising suspicion for meropenem‐induced fever. Meropenem was discontinued and replaced with ceftriaxone (2 g every 12 h), which provided adequate CNS penetrance and coverage for the *E. coli* isolate (Table 3), and metronidazole (500 mg every 8 h) for *Bacteroides fragilis* coverage.

**Table 3:** Antimicrobial susceptibility testing results for *Escherichia coli* isolated from the intraoperative abscess culture.

Antimicrobial	MIC	Susceptibility
Amox/K clavulanate	≤ 8.00 μg/mL	Susceptible
AMP/sulbactam	16/8 μg/mL	Intermediate
Ampicillin	32.00 μg/mL	Resistant
Cefazolin	4.00 μg/mL	Intermediate
Cefepime	≤ 2.00 μg/mL	Susceptible
Ceftriaxone	≤ 1.00 μg/mL	Susceptible
Cefuroxime	16.00 μg/mL	Intermediate
Ciprofloxacin	1.00 μg/mL	Resistant
Gentamicin	≤ 2.00 μg/mL	Susceptible
Levofloxacin	1.00 μg/mL	Intermediate
Minocycline	≤ 4.00 μg/mL	Susceptible
Piperacil/Tazo	≤ 8 μg/mL	Susceptible
Tetracycline	≥ 16.00 μg/mL	Resistant
Trimeth/sulfamethoxazole	≥ 4.00 μg/mL	Resistant

### 2.6. Recovery and Discharge

The EVD was removed on Hospital day 13. The patient underwent one final procedure with ENT to remove the retained intracochlear electrode for definitive source control.

On Hospital day 18, the patient was medically stable for discharge to inpatient rehabilitation with arrangements for home antibiotic infusions for a total planned duration of 6 weeks, consistent with standard recommendations for the management of intracranial abscesses in the setting of previously infected intracranial hardware.

## 3. Discussion

To our knowledge, no prior case outlines CI infection resulting in cerebellar infiltration and the rare but serious implication of cerebral herniation.

The extension of CI infection into cerebellar structures remains poorly documented. Eggink et al. note the rare occurrence of cerebellar abscess resulting from otogenic infection, secondary to chronic middle ear disease, traversing the internal auditory canal to the cerebellar peduncle, outlining a likely pathway [4]. Yet, no instances of postoperative CI infection resulting in cerebellar complications have been reported.

The retained right electrode array of the patient’s prior CI is likely the source of infection, with subsequent extension into cerebellar anatomical structures causing abscess formation. The electrode array is commonly retained to ensure the patency of the cochlear lumen, with goals for eventual reimplantation. Fortunately, studies report low microbial burden of these retained parts. However, infected intracochlear implants have a significant effect on long‐term reimplantation outcomes. Varadarajan et al. suggest that similar to cardiac implants, replaced or retained cochlear implants do pose an increased risk of secondary infection and the development of resistant organisms [7]. This was evidenced in our case, with colonization of an unusual and resistant pathogen, ESBL *E. coli*, resulting in severe infectious complications. Therefore, retained hardware should be considered when assessing risk and antibiotic choice.

Preventative measures for infectious CI complications include immunization against *Streptococcus pneumoniae* and *Haemophilus influenzae* Type B–otogenic pathogens implicated in meningitis [1]. Pressure dressings, to prevent hematoma and seroma formation, and conservative local surgical site care are additional measures used postoperatively [1]. Long‐term care includes close monitoring of external hardware for cleanliness, along with interdisciplinary follow‐up with audiology and otolaryngology.

Perioperatively, antibiotics are often recommended but vary greatly in agent, route, and duration [7, 8]. There are no current clear international guidelines for perioperative or postoperative antibiotics for CI implantation or ex‐plantation with retained arrays [8]. Despite the aforementioned low rates of adverse events, the significant complications outlined in this case may suggest an increased role of defined postoperative prophylaxis, or close surveillance for surgical management, especially in those with risk factors (retained hardware, history of otogenic or CI infections, or chronic ear pathology). Surgical removal of the retained hardware may remove the potential nidus of infection, but intervention poses its own risks; there are few studies examining the morbidity benefit, especially given the rarity of CI infection [7]. Thus, this case suggests potential benefit to electrode array removal in patients with prolonged retention and the history of recurrent infections, resistant pathogens, or poor surgical site care.

Further studies should be undertaken to evaluate additional methods to prevent retained CI hardware infections while maintaining the viability of surrounding anatomical structures to allow for reimplantation. Interestingly, Varadarajan et al. suggest the placement of an antimicrobial drug‐eluting stent to mitigate this; similar innovations must be investigated [7].

This patient’s hospitalization was further complicated by the formation of two right cerebellar abscesses. Diagnosis and management of cerebellar abscesses are more difficult than their temporal counterparts. Cerebellar abscesses tend to be multicentric, have more nuance to clinically detect, and are less responsive to drainage, contributing to their worse prognosis [9]. Thus, early clinical suspicion of abscess formation, after CI complication, and recognition of mass effect symptoms are imperative to halting life‐threatening consequences. The patient’s signs and symptoms had high suspicion for cerebellar pathology (i.e., dysmetria, ataxia, dysdiadochokinesia, and nystagmus) and mass effect (i.e., headache, altered mentation, pupillary changes, gaze preference, and irregular breathing), with imaging confirming the diagnosis.

Timely surgical intervention is the utmost priority in the management of cerebellar abscesses. Drainage, excision, aspiration, and craniectomy (in the event of elevated ICP) may all be warranted measures to provide source control. Recommendations also suggest administration of IV broad‐spectrum antibiotics for approximately 6–8 weeks with narrowing following susceptibility reports (ideally from intraoperative cultures). As mentioned, the majority of cerebellar abscesses are secondary to chronic otitis media. Otogenic causes of cerebellar abscesses include *Streptococcus, Bacteroides, Pseudomonas,* and Enterobacteriaceae species. Appropriate antimicrobials typically include metronidazole, third‐generation cephalosporins, and penicillin G [8, 10]. Coadministration of antibiotics with surgery yields the most optimal outcomes [9, 10]. Our case’s implicated organisms of *B. fragilis* and ESBL *E. coli* represent both expected and unexpected pathogens. ESBL *E. coli* is likely a product of the patient’s prior infectious colonization, necessitating multiple interventions and possible nosocomial seeding, further explaining the high likelihood and severity of complications sustained. Though steroids may reduce edema associated with mass effect and resulting herniation, their use is still controversial in the setting of intracranial abscesses. Sequelae of abscesses may include anatomically dependent neurological deficits and seizures; seizure prophylaxis with antiepileptic therapies is imperative, as seen here. Thus, this case provides a rare, detailed account and management of cerebellar complications following infection from a highly suspected CI‐related postoperative infection, shedding light on key clinical signs and the role of early appropriate intervention.

In conclusion, cerebellar abscesses leading to herniation are highly rare but notable sequelae of CI infection. The rarity of such presentation highlights a gap in clinical consensus or guidelines for postoperative antibiotic prophylaxis in CI implantation/explantation and provides an account of grave consequences likely following the retention of the electrode array with poor surgical site wound care. Despite CI having a low rate of complications, providers must keep invasive infection in adjacent areas in their differential diagnosis along with the potential for acute, life‐threatening consequences.

## Consent

Informed consent from the patient was obtained verbally on October 3rd, 2024, with two witnesses for publication of the written accounts of the events and corresponding images.

## Conflicts of Interest

The authors declare no conflicts of interest.

## Author Contributions

Authors Celine Molfetta and Ruchi Biswas contributed equally to this work.

## Funding

No funding was received for this study.

## Data Availability

All data used to support the findings of this study are included within the article.
